# Cracks in the Curriculum: The Hidden Deficiencies in Fungal Disease Coverage in Medical Books

**DOI:** 10.1093/ofid/ofaf145

**Published:** 2025-04-01

**Authors:** Isabela H Rost, Morghana M da Rosa, Sofia de O Belardinelli, Elisa H Casani, Gabriela P Macelaro, Caroline D S Giordani, Eduarda P Borsa, Ana Laura C de S. Ferreira, Larissa N Takeda, Alessandro C Pasqualotto

**Affiliations:** Federal University of Health Sciences of Porto Alegre (UFCSPA), Porto Alegre, Brazil; Federal University of Health Sciences of Porto Alegre (UFCSPA), Porto Alegre, Brazil; Federal University of Health Sciences of Porto Alegre (UFCSPA), Porto Alegre, Brazil; Federal University of Health Sciences of Porto Alegre (UFCSPA), Porto Alegre, Brazil; Federal University of Health Sciences of Porto Alegre (UFCSPA), Porto Alegre, Brazil; Federal University of Health Sciences of Porto Alegre (UFCSPA), Porto Alegre, Brazil; Federal University of Health Sciences of Porto Alegre (UFCSPA), Porto Alegre, Brazil; Federal University of Health Sciences of Porto Alegre (UFCSPA), Porto Alegre, Brazil; Federal University of Health Sciences of Porto Alegre (UFCSPA), Porto Alegre, Brazil; Federal University of Health Sciences of Porto Alegre (UFCSPA), Porto Alegre, Brazil; Santa Casa de Porto Alegre, Porto Alegre, Brazil

**Keywords:** health care professionals, medical education, mycology, teaching materials, textbooks

## Abstract

Textbooks remain a primary source of reference and education for many health care professionals and students. This study assessed the mycology content in leading internal medicine and infectious diseases textbooks, revealing significant gaps across multiple areas. Infectious diseases textbooks demonstrated better coverage compared with internal medicine textbooks, highlighting the need for improved educational resources.

Fungal infections are an increasing public health concern, causing >3.8 million deaths annually [1, 2]. Immunocompromised patients, such as those with HIV/AIDS, cancer, or organ transplants, are particularly vulnerable. The economic burden associated with the diagnosis and treatment of these infections is substantial, with global annual costs reaching billions of dollars [3].

Given the impact of these conditions, adequate training of health care professionals and graduate students is essential. Access to knowledge is now readily available online, yet many people still rely on traditional textbooks in their pursuit of information. However, the mycology content in these textbooks may be outdated, failing to incorporate advances in therapies and diagnostics [1, 4]. Therefore, it is crucial to ensure that the information conveyed aligns with the most recent data and guidelines to support accurate learning and decision-making.

This study aims to quantitatively assess the mycology content in leading internal medicine and infectious diseases textbooks. By comparing textbook content with relevant mycology studies and international guidelines, the analysis seeks to identify gaps and outdated information. The ultimate goal is to ensure that medical education remains aligned with current scientific advancements and best clinical practices.

## METHODS

We reviewed key internal medicine textbooks, including *Harrison's Principles of Internal Medicine* (2022), *Cecil Textbook of Medicine* (2023), and *Current Medical Diagnosis and Treatment* (2024), as well as the infectious diseases books *Mandell, Douglas, and Bennett's Principles and Practice of Infectious Diseases* (2019), and *Feigin and Cherry's Textbook of Pediatric Infectious Diseases* (2018).

Fungal infections were selected from the World Health Organization's Fungal Priority Pathogens List, focusing on diseases with systemic manifestations. Critical and high-priority pathogens include candidiasis, cryptococcosis, aspergillosis, mucormycosis, histoplasmosis, and mycetoma. Medium-priority pathogens like coccidioidomycosis, paracoccidioidomycosis, and talaromycosis, as well as blastomycosis, chromoblastomycosis, and sporotrichosis, were also included for their public health importance and outbreak association. The mycology content of these selected textbooks was systematically evaluated to review relevant aspects related to epidemiology, diagnosis, clinical manifestations, treatment, prognosis, and prevention of fungal diseases. The quality of the content was assessed against current medical mycology knowledge using recent publications and guidelines as benchmarks. Details of these benchmarks, along with a list of 293 topics reviewed, are provided in the Supplementary Data (Appendix A and Appendix B). The DISCERN instrument was used to evaluate content [5], with scores ranging from 1 (absent) to 5 (comprehensive).

The level of expertise in fungal diseases among textbook chapter authors was assessed by analyzing their publication history on PubMed.

Descriptive statistics were used to summarize the data. Qualitative variables were compared using chi-square tests, while Kruskal-Wallis tests evaluated score variance between textbooks on a pairwise basis. Statistical analysis was performed using SPSS 22.0. Statistical significance was determined for *P* values <.05.

## RESULTS

**Figure 1.**
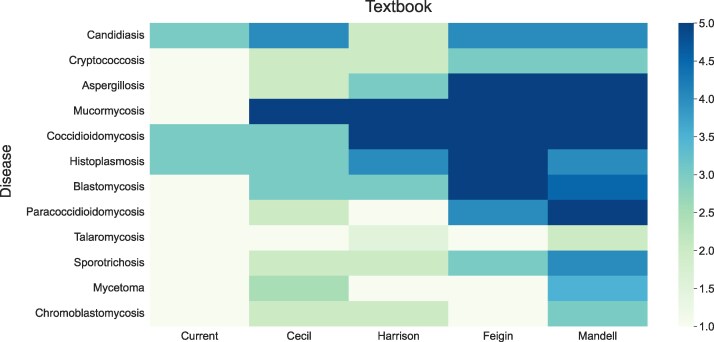
Comparison of fungal disease coverage across key medical textbooks. Each average score reflects the level of detail dedicated to a specific fungal disease in each textbook. A higher score indicates more comprehensive coverage.

**Figure 2.**
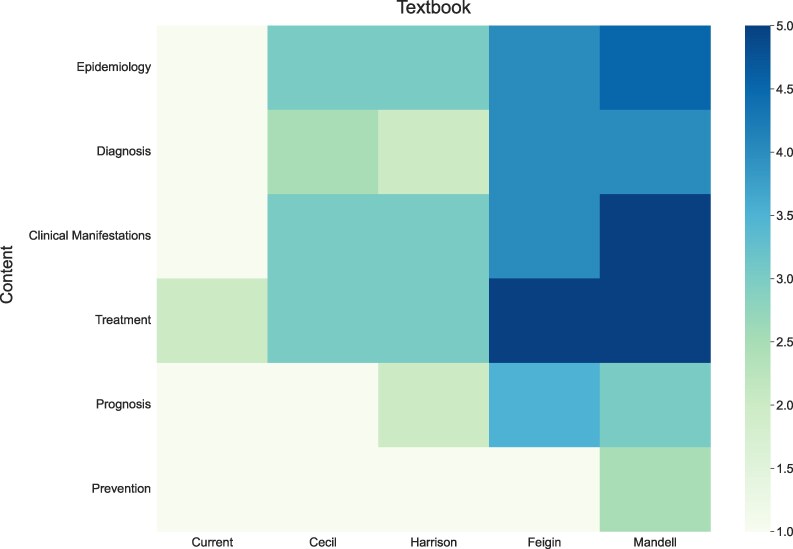
Specific content related to fungal diseases covered by key medical textbooks. Each average score reflects the level of detail dedicated to a specific topic in each textbook. A higher score indicates more comprehensive coverage.

Figure 1 illustrates the extent of fungal disease coverage in each book. *Mandell* provided comprehensive coverage, achieving scores ≥4 for 66.7% of the diseases. However, it showed weaker coverage for cryptococcosis, chromoblastomycosis (median, 3 each), and talaromycosis (median, 2). As shown in Figure 2, which analyzes coverage by topic, *Mandell* excelled overall, with one-third of the topics achieving a median of 5.

*Feigin* provided detailed content on aspergillosis, mucormycosis, histoplasmosis, blastomycosis, and coccidioidomycosis (median, 5 each). Coverage was balanced overall, with most conditions scoring highly, except talaromycosis, chromoblastomycosis, and mycetoma (median, 1). Most topics received a median of 4 or higher, except for prognosis (median, 3.5) and prevention (median, 1).

*Harrison* excelled in mucormycosis and coccidioidomycosis (median, 5 each) but underperformed in 75% of fungal diseases, with a median of 3 or less. It had the lowest score among all textbooks for candidosis (median, 2), also performing poorly for paracoccidioidomycosis and mycetoma (median, 1). Regarding specific topics, no area had a median above 3, with particularly weak coverage in diagnosis (median, 2) and prevention (median, 1).

*Cecil* provided excellent coverage for mucormycosis (median, 5) and performed well on candidosis (median, 4). Its coverage was limited for most fungal diseases, including aspergillosis, cryptococcosis, sporotrichosis, paracoccidioidomycosis, and chromoblastomycosis (median, 2 each). The median for talaromycosis was 1. *Cecil* showed a median of ≤3 for all topics evaluated.

*Current* had poor overall coverage, with 75% of the evaluated fungal diseases receiving a median score of 1. The best scores in *Current* occurred for candidosis, histoplasmosis, and coccidioidomycosis (median, 3 each). *Current* had the worst performance among evaluated textbooks in coverage of epidemiology, diagnosis, clinical manifestations, and treatment, with 83.3% of topics scoring a median of 1.

Statistical analyses showed significant variability in disease coverage. Post hoc analyses confirmed that *Mandell* and *Feigin* consistently outperformed internal medicine textbooks, with statistically significant differences (*P* < .05) for most diseases in pairwise comparisons, including aspergillosis, paracoccidioidomycosis, and mucormycosis. *Harrison* and *Cecil* displayed more variable coverage, generally offering intermediate performance across most diseases and topics. *Current* consistently underperformed, particularly in prevention and diagnosis, scoring significantly lower than other textbooks (*P* < .05 for most fungal diseases).

Among the textbooks evaluated in this study, *Current* was the only one with mycology chapters authored by individuals who were not key opinion leaders in the field of medical mycology.

## DISCUSSION

The comparison of internal medicine and infectious diseases textbooks revealed that the latter provided more specialized coverage of mycology. This finding aligns with previous studies analyzing medical books on other specialties, making this study innovative in mycology [6, 7]. On diagnosis, treatment, and prognosis, internal medicine books received the lowest ratings, revealing limited or outdated coverage. For example, *Harrison* presented inadequate coverage in 75% of the diseases and in all topics. Therefore, this lack of information may compromise the education of students and the updates of professionals [8]. These data support the hypothesis of this study that infectious diseases books are more suitable for the teaching and practice of fungal infectious diseases, offering more detailed and specific information.

Concerning clinical manifestations of fungal diseases, all internal medicine books received a median of 3.0 or less, which is concerning given the importance of this information for general practitioners. Identifying clinical manifestations is a crucial part of the early recognition of mycoses, enabling the prompt initiation of antifungal therapy—both essential factors for effective patient management [9].

The predominance of infectious disease specialists as chapter authors was notable across most evaluated texts. *Current*, however, was authored by a less specialized team, which may contribute to its lowest coverage compared with other texts.

The study found a disproportionate emphasis on epidemiology rather than prevention, as the latter received a median of 1 in all textbooks, except for *Mandell* (median, 2.5), a troubling factor in light of the increasing role of preventive measures in clinical settings, especially for high-risk groups [1, 10, 11]. *Mandell*, which scored highest in prevention, still had limited coverage in the area, indicating opportunities for potential development.

Several important topics were missing across all books, including the link between histoplasmosis and coinfections and the inclusion of emerging fungal species [12]. Critical gaps included the impact of COVID-19 on fungal diseases and the use of antifungal prophylaxis in high-risk populations, as well as propensity scores for candidemia [13]. Specific aspects of paracoccidioidomycosis and sporotrichosis management were also omitted [14, 15]. Concerning mycetoma, none of the evaluated books showed information on prognosis and prevention, a disease with very difficult treatment [16]. Besides, *Current* and *Feigin* had very little content on chromoblastomycosis, an important mycotic disease [17]. Talaromycosis and cryptococcosis were also under-represented [18–20].

As fungal diseases may present nonspecific symptoms and require specific diagnoses and treatments [21], the lack of adequate information can lead to misdiagnosis and inadequate therapies, increasing mortality risk. This underestimation of relevance also impacts health policies [22]. The need for constant updating in managing fungal infections is widely recognized, especially as new strains of multiresistant fungi are becoming more prevalent. It is important to highlight that textbooks often take years to be revised, making it challenging for them to remain up to date with recent advancements. This delay might explain why certain significant topics were inadequately covered, which is particularly critical for rapidly evolving fields like medical mycology.

One of the study's limitations is the small number of textbooks analyzed, although the texts we used are among the most established and widely used resources in medical education. Some aspects of fungal diseases were not fully covered, including superficial infections and specific high-risk populations like burn patients. This limitation reduced the comprehensiveness of the analysis by not covering the full spectrum of fungal diseases and their clinical implications. However, the focus was intentionally placed on systemic fungal infections, which are more complex and critical for medical training.

To ensure that health care professionals are educated to handle the complexity of fungal infections in today's context, it is essential for reference materials to reflect best practices based on evidence, new discoveries, and the latest guidelines. Considering the gaps in textbooks, relying on up-to-date guidelines and peer-reviewed articles can enhance medical education by providing more accurate and current information. This approach supports the provision of high-quality, safe, and effective patient care, minimizing risks and optimizing clinical outcomes.

## Supplementary Material

ofaf145_Supplementary_Data

## Supplementary Data

Supplementary materials are available at *Open Forum Infectious Diseases* online. Consisting of data provided by the authors to benefit the reader, the posted materials are not copyedited and are the sole responsibility of the authors, so questions or comments should be addressed to the corresponding author.
