# Supplementary material for: Cracks in the Curriculum: The Hidden Deficiencies in Fungal Disease Coverage in Medical Books
Source: Open Forum Infect Dis. 2025 Apr 1;12(4):ofaf145. doi: 10.1093/ofid/ofaf145 (PMC11957914; doi:10.1093/ofid/ofaf145)
Supplement: ofaf145_Supplementary_Data [file ofaf145_supplementary_data.docx]

**Supplemental materials for:**

**Cracks in the curriculum: the hidden deficiencies in fungal disease coverage in medical books**

# **Appendix A – Selected guidelines and articles**

The mycology content in these books was evaluated based on epidemiology, diagnosis, treatment, and prevention, using relevant guidelines and studies published in high-impact journals over the last 10 years. To ensure quality, only studies from rigorous peer-reviewed journals were included. Preference was given to guidelines from leading medical societies to assess the coverage and quality of information in medical mycology. The selected articles are listed below.

J Michael Miller, Matthew J Binnicker, Sheldon Campbell, Karen C Carroll, Kimberle C Chapin, Mark D Gonzalez, Amanda Harrington, Robert C Jerris, Sue C Kehl, Sixto M Leal, Robin Patel, Bobbi S Pritt, Sandra S Richter, Barbara Robinson-Dunn, James W Snyder, Sam Telford, Elitza S Theel, Richard B Thomson, Melvin P Weinstein, Joseph D Yao, Guide to Utilization of the Microbiology Laboratory for Diagnosis of Infectious Diseases: 2024 Update by the Infectious Diseases Society of America (IDSA) and the American Society for Microbiology (ASM) , Clinical Infectious Diseases, 2024; <https://doi.org/10.1093/cid/ciae104>

John N. Galgiani, Neil M. Ampel, Janis E. Blair, Antonino Catanzaro, Francesca Geertsma, Susan E. Hoover, Royce H. Johnson, Shimon Kusne, Jeffrey Lisse, Joel D. MacDonald, Shari L. Meyerson, Patricia B. Raksin, John Siever, David A. Stevens, Rebecca Sunenshine, Nicholas Theodore, 2016 Infectious Diseases Society of America (IDSA) Clinical Practice Guideline for the Treatment of Coccidioidomycosis, Clinical Infectious Diseases, Volume 63, Issue 6, 15 September 2016, Pages e112–e146, <https://doi.org/10.1093/cid/ciw360>

Thomas F. Patterson, George R. Thompson, David W. Denning, Jay A. Fishman, Susan Hadley, Raoul Herbrecht, Dimitrios P. Kontoyiannis, Kieren A. Marr, Vicki A. Morrison, M. Hong Nguyen, Brahm H. Segal, William J. Steinbach, David A. Stevens, Thomas J. Walsh, John R. Wingard, Jo-Anne H. Young, John E. Bennett, Practice Guidelines for the Diagnosis and Management of Aspergillosis: 2016 Update by the Infectious Diseases Society of America, Clinical Infectious Diseases, Volume 63, Issue 4, 15 August 2016, Pages e1–e60, https://doi.org/10.1093/cid/ciw326

Peter G. Pappas, Carol A. Kauffman, David R. Andes, Cornelius J. Clancy, Kieren A. Marr, Luis Ostrosky-Zeichner, Annette C. Reboli, Mindy G. Schuster, Jose A. Vazquez, Thomas J. Walsh, Theoklis E. Zaoutis, Jack D. Sobel, Clinical Practice Guideline for the Management of Candidiasis: 2016 Update by the Infectious Diseases Society of America, Clinical Infectious Diseases, Volume 62, Issue 4, 15 February 2016, Pages e1–e50, https://doi.org/10.1093/cid/civ933J

David W. Denning Jacques Cadranel Catherine Beigelman-Aubry Florence Ader Arunaloke Chakrabarti Stijn Blot Andrew J. Ullmann George Dimopoulos Christoph Lange, Chronic pulmonary aspergillosis: rationale and clinical guidelines for diagnosis and management, European Respiratory Journal 2015; https://doi.org/10.1183/13993003.00583-2015

Martin-Loeches I, Antonelli M, Cuenca-Estrella M, et al. ESICM/ESCMID task force on practical management of invasive candidiasis in critically ill patients. Intensive Care Med. 2019;45(6):789-805. [https://doi.org/](https://doi.org/10.1093/cid/ciae104)10.1007/s00134-019-05599-w

Hoenigl M, Salmanton-García J, Walsh TJ, Nucci M, Neoh CF, Jenks JD, Lackner M, Sprute R, Al-Hatmi AMS, Bassetti M, Carlesse F, Freiberger T, Koehler P, Lehrnbecher T, Kumar A, Prattes J, Richardson M, Revankar S, Slavin MA, Stemler J, Spiess B, Taj-Aldeen SJ, Warris A, Woo PCY, Young JH, Albus K, Arenz D, Arsic-Arsenijevic V, Bouchara JP, Chinniah TR, Chowdhary A, de Hoog GS, Dimopoulos G, Duarte RF, Hamal P, Meis JF, Mfinanga S, Queiroz-Telles F, Patterson TF, Rahav G, Rogers TR, Rotstein C, Wahyuningsih R, Seidel D, Cornely OA. Global guideline for the diagnosis and management of rare mould infections: an initiative of the European Confederation of Medical Mycology in cooperation with the International Society for Human and Animal Mycology and the American Society for Microbiology. Lancet Infect Dis. 2021 Aug;21(8):e246-e257. doi: 10.1016/S1473-3099(20)30784-2. Epub 2021 Feb 16. Erratum in: Lancet Infect Dis. 2021 Apr;21(4):e81. [https://doi.org/](https://doi.org/10.1093/cid/ciae104)10.1016/S1473-3099(21)00124-9. PMID: 33606997.

Hage CA, Carmona EM, Epelbaum O, Evans SE, Gabe LM, Haydour Q, Knox KS, Kolls JK, Murad MH, Wengenack NL, Limper AH. Microbiological Laboratory Testing in the Diagnosis of Fungal Infections in Pulmonary and Critical Care Practice. An Official American Thoracic Society Clinical Practice Guideline. Am J Respir Crit Care Med. 2019 Sep 1;200(5):535-550. doi: 10.1164/rccm.201906-1185ST. Erratum in: Am J Respir Crit Care Med. 2019 Nov 15;200(10):1326. [https://doi.org/](https://doi.org/10.1093/cid/ciae104)10.1164/rccm.v200erratum8. PMID: 31469325; PMCID: PMC6727169.

Husain S, Sole A, Alexander BD, Aslam S, Avery R, Benden C, Billaud EM, Chambers D, Danziger-Isakov L, Fedson S, Gould K, Gregson A, Grossi P, Hadjiliadis D, Hopkins P, Luong ML, Marriott DJE, Monforte V, Muñoz P, Pasqualotto AC, Roman A, Silveira FP, Teuteberg J, Weigt S, Zaas AK, Zuckerman A, Morrissey O. The 2015 International Society for Heart and Lung Transplantation Guidelines for the management of fungal infections in mechanical circulatory support and cardiothoracic organ transplant recipients: Executive summary. he Journal of Heart and Lung Transplantation, v. 35, n. 3, p. 261–282, mar. 2016. [https://doi.org/](https://doi.org/10.1093/cid/ciae104)10.1016/j.healun.2016.01.007. Epub 2016 Jan 19. PMID: 26970469.

Guidelines for diagnosing, preventing and managing cryptococcal disease among adults, adolescents and children living with HIV. Genebra, Switzerland: World Health Organization, 2022. https://iris.who.int/bitstream/handle/10665/360423/9789240053298-eng.pdf?sequence=1

Guidelines for diagnosing and managing disseminated histoplasmosis among people living with HIV. Washington, D.C.: Pan American Health Organization, World Health Organization; 2020 <https://iris.paho.org/bitstream/handle/10665.2/52304/9789275122495_eng.pdf>

Groll AH, Castagnola E, Cesaro S, et al. Fourth European Conference on Infections in Leukaemia (ECIL-4): guidelines for diagnosis, prevention, and treatment of invasive fungal diseases in paediatric patients with cancer or allogeneic haemopoietic stem-cell transplantation. Lancet Oncol. 2014;15(8):e327-e340. [https://doi.org/](https://doi.org/10.1093/cid/ciae104)10.1016/S1470-2045(14)70017-8

Tissot F, Agrawal S, Pagano L, et al. ECIL-6 guidelines for the treatment of invasive candidiasis, aspergillosis and mucormycosis in leukemia and hematopoietic stem cell transplant patients. Haematologica. 2017;102(3):433-444. [https://doi.org/](https://doi.org/10.1093/cid/ciae104)10.3324/haematol.2016.152900

Groll AH, Pana D, Lanternier F, et al. 8th European Conference on Infections in Leukaemia: 2020 guidelines for the diagnosis, prevention, and treatment of invasive fungal diseases in paediatric patients with cancer or post-haematopoietic cell transplantation. Lancet Oncol. 2021;22(6):e254-e269. [https://doi.org/](https://doi.org/10.1093/cid/ciae104)10.1016/S1470-2045(20)30723-3

Colombo AL, Guimarães T, Camargo LF, et al. Brazilian guidelines for the management of candidiasis - a joint meeting report of three medical societies: Sociedade Brasileira de Infectologia, Sociedade Paulista de Infectologia and Sociedade Brasileira de Medicina Tropical. Braz J Infect Dis. 2013;17(3):283-312. [https://doi.org/](https://doi.org/10.1093/cid/ciae104)10.1016/j.bjid.2013.02.001

Cornely OA, Alastruey-Izquierdo A, Arenz D, et al. Global guideline for the diagnosis and management of mucormycosis: an initiative of the European Confederation of Medical Mycology in cooperation with the Mycoses Study Group Education and Research Consortium. Lancet Infect Dis. 2019 Dec;19(12):e405-e421. [https://doi.org/](https://doi.org/10.1093/cid/ciae104)10.1016/S1473-3099(19)30312-3. Epub 2019 Nov 5. PMID: 31699664; PMCID: PMC8559573.

Galgiani JN, Ampel NM, Blair JE, Catanzaro A, Geertsma F, Hoover SE, Johnson RH, Kusne S, Lisse J, MacDonald JD, Meyerson SL, Raksin PB, Siever J, Stevens DA, Sunenshine R, Theodore N. 2016 Infectious Diseases Society of America (IDSA) Clinical Practice Guideline for the Treatment of Coccidioidomycosis. Clin Infect Dis. 2016 Sep 15;63(6):e112-46. [https://doi.org/](https://doi.org/10.1093/cid/ciae104)10.1093/cid/ciw360. PMID: 27470238.

Cooksey GS, Nguyen A, Knutson K, et al. Notes from the Field: Increase in Coccidioidomycosis — California, 2016. MMWR Morb Mortal Wkly Rep 2017;66:833–834. DOI: <http://dx.doi.org/10.15585/mmwr.mm6631a4>

Herrick KR, Trondle ME, Febles TT. Coccidioidomycosis (Valley Fever) in Primary Care. Am Fam Physician. 2020 Feb 15;101(4):221-228. PMID: 32053327. https://www.aafp.org/pubs/afp/issues/2020/0215/p221.pdf

Fisher, F. S., Bultman, M. W., & Pappagianis, D. (2000). Operational guidelines (version 1.0) for geological fieldwork in areas endemic for Coccidioidomycosis (Valley Fever). Em Open-File Report (Version 1.0). US Geological Survey. https://doi.org/10.3133/ofr00348

Viale MN, Caceres DH, Mansilla PE, Lopez-Joffre MC, Vivot FG, Motter AN, Toranzo AI, Canteros CE. Evaluation of the Analytical Performance of a Lateral Flow Assay for the Detection of Anti-Coccidioides Antibodies in Human Sera—Argentina. Journal of Fungi. 2024; 10(5):322. <https://doi.org/10.3390/jof10050322>

Grill FJ, Jugler C, Kaleta E, Chen Q, Magee DM, Grys TE, Lake DF. Clinical Laboratory Utility of a Humanized Antibody in Commercially Available Enzyme Immunoassays for Coccidioidomycosis. Microbiol Spectr. 2022 Oct 26;10(5):e0257322. [https://doi.org/](https://doi.org/10.3390/jof10050322)10.1128/spectrum.02573-22. Epub 2022 Sep 19. PMID: 36121238; PMCID: PMC9602258.

Stanley W. Chapman, William E. Dismukes, Laurie A. Proia, Robert W. Bradsher, Peter G. Pappas, Michael G. Threlkeld, Carol A. Kauffman, Clinical Practice Guidelines for the Management of Blastomycosis: 2008 Update by the Infectious Diseases Society of America, Clinical Infectious Diseases, Volume 46, Issue 12, 15 June 2008, Pages 1801–1812, <https://doi.org/10.1086/588300>

Peçanha PM, Peçanha-Pietrobom PM, Grão-Velloso TR, Rosa Júnior M, Falqueto A, Gonçalves SS. Paracoccidioidomycosis: What We Know and What Is New in Epidemiology, Diagnosis, and Treatment. Journal of Fungi. 2022; 8(10):1098. https://doi.org/10.3390/jof8101098

Thompson GR 3rd, Le T, Chindamporn A, et al. Global guideline for the diagnosis and management of the endemic mycoses: an initiative of the European Confederation of Medical Mycology in cooperation with the International Society for Human and Animal Mycology. The Lancet. Infectious Diseases. 2021 Dec;21(12):e364-e374. [https://doi.org/](https://doi.org/10.3390/jof10050322)10.1016/s1473-3099(21)00191-2. PMID: 34364529; PMCID: PMC9450022.

Pasqualotto AC, ed. Endemic mycoses: recent advances in epidemiology, diagnosis and treatment. J Fungi. 2024 Jul;10(7):1-196.https://doi.org/10.3390/books978-3-7258-1444-2.

Donnelly JP, Chen SC, Kauffman CA, Steinbach WJ, Baddley JW, Verweij PE, Clancy CJ, Wingard JR, Lockhart SR, Groll AH, Sorrell TC, Bassetti M, Akan H, Alexander BD, Andes D, Azoulay E, Bialek R, Bradsher RW, Bretagne S, Calandra T, Caliendo AM, Castagnola E, Cruciani M, Cuenca-Estrella M, Decker CF, Desai SR, Fisher B, Harrison T, Heussel CP, Jensen HE, Kibbler CC, Kontoyiannis DP, Kullberg BJ, Lagrou K, Lamoth F, Lehrnbecher T, Loeffler J, Lortholary O, Maertens J, Marchetti O, Marr KA, Masur H, Meis JF, Morrisey CO, Nucci M, Ostrosky-Zeichner L, Pagano L, Patterson TF, Perfect JR, Racil Z, Roilides E, Ruhnke M, Prokop CS, Shoham S, Slavin MA, Stevens DA, Thompson GR, Vazquez JA, Viscoli C, Walsh TJ, Warris A, Wheat LJ, White PL, Zaoutis TE, Pappas PG. Revision and Update of the Consensus Definitions of Invasive Fungal Disease From the European Organization for Research and Treatment of Cancer and the Mycoses Study Group Education and Research Consortium. Clin Infect Dis. 2020 Sep 12;71(6):1367-1376. [https://doi.org/](https://doi.org/10.3390/books978-3-7258-1444-2)10.1093/cid/ciz1008. PMID: 31802125; PMCID: PMC7486838.

Lehrnbecher T, Fisher BT, Phillips B, Beauchemin M, Carlesse F, Castagnola E, Duong N, Dupuis LL, Fioravantti V, Groll AH, Haeusler GM, Roilides E, Science M, Steinbach WJ, Tissing W, Warris A, Patel P, Robinson PD, Sung L. Clinical Practice Guideline for Systemic Antifungal Prophylaxis in Pediatric Patients With Cancer and Hematopoietic Stem-Cell Transplantation Recipients. J Clin Oncol. 2020 Sep 20;38(27):3205-3216. [https://doi.org/](https://doi.org/10.3390/books978-3-7258-1444-2)10.1200/JCO.20.00158. PMID: 32459599; PMCID: PMC7499615.

Orofino-Costa R, Freitas DFS, Bernardes-Engemann AR, Rodrigues AM, Talhari C, Ferraz CE, Veasey JV, Quintella L, Sousa MSLA, Vettorato R, Almeida-Paes R, de Macedo PM. Human sporotrichosis: recommendations from the Brazilian Society of Dermatology for the clinical, diagnostic and therapeutic management. An Bras Dermatol. 2022 Nov-Dec;97(6):757-777. [https://doi.org/](https://doi.org/10.3390/books978-3-7258-1444-2)10.1016/j.abd.2022.07.001. PMID: 36155712; PMCID: PMC9582924.

Perfect JR, Dismukes WE, Dromer F, Goldman DL, Graybill JR, Hamill RJ, Harrison TS, Larsen RA, Lortholary O, Nguyen MH, Pappas PG, Powderly WG, Singh N, Sobel JD, Sorrell TC. Clinical practice guidelines for the management of cryptococcal disease: 2010 update by the infectious diseases society of america. Clin Infect Dis. 2010 Feb 1;50(3):291-322. [https://doi.org/](https://doi.org/10.3390/books978-3-7258-1444-2)10.1086/649858. PMID: 20047480; PMCID: PMC5826644.

# **Appendix B – Detailed Scoring of Subtopics for Fungal Diseases**

| **Mycosis** | **Topic** | **Subtopic** |
| --- | --- | --- |
| **A**  **s**  **p**  **e**  **r**  **g**  **i**  **l**  **l**  **o**  **s**  **i**  **s** | Epidemiology | Risk factors beyond neutropenia (COVID, Influenza, diabetes, COPD, new immunomodulatory agents) – emerging populations for aspergillosis |
|  |  | Cryptic Aspergillus species (importance, differences in drug susceptibility, speciation tests, differences in complexes) |
|  |  | Epidemiology of resistance |
|  |  | Epidemiology of allergic bronchopulmonary aspergillosis (mainly asthmatics and cystic fibrosis) and severe asthma with fungal sensitivity |
|  |  | Epidemiology of chronic cavitary pulmonary aspergillosis |
|  | Clinical manifestations | Clinical forms and different syndromes (chronic: APBA, SAFS, CCPA, semi-invasive aspergillosis, IPA, CAPA/IAPA), and overlap between syndromes |
|  |  | Description of clinical forms of aspergillosis involving paranasal sinuses (invasive form, fungal ball, and allergic form) |
|  |  | Clinical presentation of disseminated aspergillosis |
|  |  | Aspergillosis occurring after surgeries – when to suspect, at least mention its existence |
|  | Diagnosis | Radiological diagnosis (CT, PET-CT, other methods) |
|  |  | Antigen test: galactomannan (performance in neutropenic versus non-neutropenic patients, serum versus other specimens including BAL, discussion of the optimal cutoff point for BAL, treatment monitoring with antigen – tracking and prognosis, testing in patients on prophylaxis for filamentous fungi such as posaconazole) |
|  |  | How to differentiate colonization and infection |
|  |  | How to optimize culture results - use of greater volume, culture media to be used, with antibiotics to inhibit bacterial growth |
|  |  | The role of serology in chronic forms |
|  |  | Strategies for differentiating syndromes that look like tuberculosis |
|  |  | Allergy skin tests for APBA/SAFS, and cut-off points for total IgE and specific IgE against Aspergillus |
|  | Treatment | Spectrum of drugs from different classes, such as Azoles (voriconazole, Isavuconazole - first line) and Polyenes (amphotericin B) |
|  |  | Problems with the use of voriconazole: toxicity, need to measure plasma levels (and consequences of high or low levels), unpredictable metabolism, difficulties in obtaining adequate levels in children |
|  |  | Drug interactions (Itraconazole - drugs that increase pH, Azolics in general: CYP enzymes, food) |
|  |  | Therapeutic monitoring not only of voriconazole, but also of other azoles, especially itraconazole and posaconazole. |
|  |  | Low absorption of itraconazole capsules or posaconazole oral solution. Strategies to increase absorption |
|  |  | Correction and PK in obese people, renal failure and liver failure |
|  |  | Time of therapy and radiological control |
|  |  | Indication of antifungal in APBA and treatment time |
|  |  | Antifungal in chronic forms, such as chronic cavitary pulmonary aspergillosis and rhinosinusitis |
|  |  | Embolization in cases of bleeding (hemoptysis) and surgical treatment of chronic cavitary pulmonary aspergillosis. |
|  |  | Resistance in Aspergillus - two main mechanisms: use of azoles in agriculture and exposure to long periods of antifungal drugs in chronic forms. What are the therapeutic options, how and when to look for them? |
|  | Prognosis | Prognostic factors in hematology (type of transplant, chemotherapy with greater risk, neutropenia time), in solid organ and bone marrow transplants |
|  | Prevention | HEPA filters and environmental control |
|  |  | Primary prophylaxis - when? Which agents? Prophylaxis in hematology / bone marrow transplant / solid organ transplant patients |
|  |  | Secondary prophylaxis - when to use and when to discontinue? Which agents? |
| **H**  **i**  **s**  **t**  **o**  **p**  **l**  **a**  **s**  **m**  **o**  **s**  **i**  **s** | Epidemiology | Expansion of endemic regions: Africa and Asia. Histoplasmosis as a global disease |
|  |  | Transmission mechanisms: contaminated soil, bats / caves, river slopes, association with watersheds |
|  |  | Impact on AIDS patients (Latin America and Africa) and occurrence in other immunosuppressions |
|  | Clinical manifestations | Different clinical forms and their cardinal symptoms. Acute pulmonary, chronic pulmonary and disseminated forms. |
|  |  | Association between inoculum and more severe acute disease |
|  |  | Mediastinal complications: adenitis, granuloma and fibrosis |
|  |  | Association with co-infections (particularly tuberculosis) and comorbidities: changes in the disease's natural history." |
|  |  | Reactivation disease vs. reinfection (immunosuppression) |
|  | Diagnosis | The role of serology and its limitations in immunosuppressed patients |
|  |  | Antigen detection as the main diagnostic tool in disseminated disease: performance in the different clinical forms. Use of LFA (lateral flow assay) |
|  |  | Difficulties with culture (time for growth) and histopathology |
|  |  | Use of PCR |
|  |  | Diagnosis of CNS disease |
|  | Treatment | Indication of itraconazole vs. amphotericin B (mild and moderate/severe forms) |
|  |  | Superiority of liposomal amphotericin. Use of single, high-dose liposomal amphotericin B (10 mg/kg) |
|  |  | Evaluation of treatment response with antigen or PCR |
|  |  | Length of maintenance therapy in immunosuppressed individuals |
|  |  | Use of other azoles such as isavuconazole or posaconazole in histoplasmosis |
|  | Prognosis | Mortality in disseminated disease, differences between hosts and regions of the world |
|  |  | The role of antigens in prognosis |
|  | Prevention | Environmental control measures in hyperendemic areas |
|  |  | Personal protective equipment in risky activities, such as cave trekking |
|  |  | Primary prophylaxis in hyperendemic regions (CD4<150) |
| **B**  **l**  **a**  **s**  **t**  **o**  **m**  **y**  **c**  **o**  **s**  **i**  **s** | Epidemiology | Geographical distribution and expansion of endemic areas |
|  |  | Risk and vulnerability factors, including relative immunosuppression such as diabetes |
|  |  | Outbreaks and environmental factors |
|  |  | Late diagnosis and underdiagnosis |
|  |  | Impact of the COVID-19 pandemic on epidemiology |
|  | Clinical manifestations | Difference in disease presentation within immunosuppressed patients |
|  |  | Extrapulmonary dissemination, including CNS disease |
|  |  | Cutaneous forms |
|  |  | Differential diagnosis in community-acquired pneumonia |
|  | Diagnosis | Role of urinary antigen |
|  |  | Bronchoalveolar lavage and culture yield indication |
|  |  | Histopathological diagnosis with GMS or PAS |
|  | Treatment | Use of liposomal amphotericin B and deoxycholate |
|  |  | Choice of azole and duration of maintenance treatment |
|  |  | Challenges in the treatment of CNS blastomycosis |
|  |  | Combination therapy / new antifungals |
|  | Prognosis | Impact of disease severity on prognosis |
|  |  | Response to treatment with itraconazole |
|  |  | Prognosis in immunocompromised patients |
|  | Prevention | Minimizing exposure |
|  |  | Early clinical recognition |
|  |  | Azole prophylaxis in selected individuals |
| **C**  **a**  **n**  **d**  **i**  **d**  **i**  **a**  **s**  **i**  **s** | Epidemiology | Importance of knowing which Candida species is causing the infection |
|  |  | expected sensitivity profile according to species |
|  |  | incidence of Candida in different settings: hematology, intensive care and surgery |
|  | Clinical manifestations | risk factors for candidemia |
|  |  | propensity scores for candidemia |
|  |  | clinical presentation of oral, esophageal, candidemia and disseminated candidiasis to different sites, such as the eye, heart valves and CNS |
|  | Diagnosis | Growth time in culture |
|  |  | Sensitivity of detection in blood culture |
|  |  | Performance of candida cultivation in different blood culture bottles |
|  |  | significance of growth in sites such as urine, skin, intra-abd secretion and lung - colonization vs. infection |
|  |  | mention of the use of selective culture media |
|  |  | performance of the beta-glucan test in cadidemia and other non-culture-based technologies, such as T2 and PCR |
|  | Treatment | echinocandins as first-line drugs for candidemia |
|  |  | difference between first and second generation echinocandins (rezafungin) |
|  |  | Importance of removing the catheter in candidemia |
|  |  | Fundus examination in candidemia |
|  |  | Is a systematic echocardiogram necessary in candidemia? |
|  |  | Empirical antimicrobial therapy in candidemia in critically ill patients - evidence and recommendations |
|  |  | Starting empirical anti-Candida therapy in febrile neutropenics |
|  |  | Importance of de-escalating antifungal therapy once the species is known and the patient is clinically stabilized |
|  |  | Penetration of antifungals into sites such as the eye, urine and intra-abdominal abscesses |
|  |  | Indications for polyenes and azoles in systemic Candida infections |
|  | Prognosis | Prognostic factors in candidemia |
|  | Prevention | Prevention of candidemia |
|  |  | Antifungal prophylaxis studies in vulnerable populations, including surgical patients and low birth weight neonates |
|  |  | Prophylaxis in hematological / bone marrow transplant / solid organ transplant patients |
| **P**  **a**  **r**  **a**  **c**  **o**  **c**  **c**  **i**  **d**  **i**  **o**  **i**  **d**  **o**  **m**  **y**  **c**  **o**  **s**  **i**  **s** | Epidemiology | Geographical distribution in Latin America |
|  |  | Epidemiology of P. lutzii |
|  |  | Association with certain agricultural practices |
|  |  | Epidemiological changes: decrease in the occurrence of the disease with the mechanization of agriculture. |
|  |  | Typical hosts in chronic forms and association with other risk factors |
|  |  | Social determinants of the disease |
|  | Clinical manifestations | Differentiation between acute and chronic forms, relationship with the host |
|  |  | Paracocci simulating neoplasia or co-existing with cancer |
|  |  | Associated immunological phenomena - immune reconstitution/paradoxical reaction |
|  |  | Differentiation between paracocci and tuberculosis |
|  |  | Disseminated forms in immunosuppressed patients, especially AIDS |
|  | Diagnosis | The role of serology and different methods |
|  |  | Serology as a control of cure |
|  |  | Limitations of classical methods (direct microscopy and culture) |
|  |  | New methods, including PCR |
|  | Treatment | Itraconazole and alternative azole drugs (voriconazole, posaconazole) |
|  |  | Role of SMX-TMP (bactrim) |
|  |  | Treatment of CNS disease |
|  |  | Duration of treatment. |
|  | Prognosis | Mortality in disseminated disease (inc. CNS) |
|  |  | Lung and skin/mucosal sequelae |
|  | Prevention | Control measures in hyperendemic/rural areas |
|  |  | Education on personal protection measures |
| **C**  **o**  **c**  **c**  **i**  **d**  **i**  **o**  **i**  **d**  **i**  **o**  **m**  **y**  **c**  **o**  **s**  **i**  **s** | Epidemiology | New endemic areas, including South America. Expansion of geographical areas |
|  |  | Cocci outbreaks around the world, environmental factors |
|  |  | Influence of climate change on the expansion of areas |
|  |  | Risk factors: exposure to soil, sandstorms, construction, hunting wild animals (armadillo) |
|  |  | Risk groups for disseminated disease |
|  | Clinical manifestations | Valley Fever: characterization |
|  |  | Frequency of symptomatic disease/primary lung disease |
|  |  | Disseminated disease, including osteoarticular, cutaneous and CNS |
|  |  | Cocci exacerbating previous respiratory disease (COPD) |
|  | Diagnosis | Role of serology, culture and other tests (CNS antigen detection) |
|  |  | Differential diagnosis in acute respiratory syndromes. When to think about cocci in community-acquired pneumonia |
|  |  | When should lumbar puncture be indicated? |
|  | Treatment | Indication of antifungal treatment? |
|  |  | Surgical treatment of cavitary disease |
|  |  | Management of CNS disease |
|  |  | Treatment in people living with HIV, transplant patients and other immunosuppressive diseases |
|  | Prognosis | Cure and recurrence rates in the different syndromes |
|  |  | Pulmonary sequelae |
|  | Prevention | Educational measures |
|  |  | Pre-emptive therapy in endemic areas (transplantation and immunobiologics) |
|  |  | Prevention and management of exposures in the laboratory environment |
| **M**  **u**  **c**  **o**  **r**  **m**  **y**  **c**  **o**  **s**  **i**  **s** | Epidemiology | Impact of COVID-19 on the incidence of mucormycosis, as in India |
|  |  | Geographical distribution and regional variations |
|  |  | Demographic and clinical risk factors: most common hosts |
|  |  | New fungal species causing mucormycosis |
|  |  | In-hospital outbreaks |
|  |  | Epidemiology of mucormycosis in hematological and transplant patients. Association between mucormycosis and decompensated diabetes (ketoacidosis) |
|  | Clinical manifestations | Different clinical forms, frequency according to host (rhinocerebral, pulmonary, cutaneous and gastrointestinal forms) |
|  |  | Differential diagnosis with aspergillosis and other hyalohyphomycosis agents |
|  |  | Atypical manifestations, when suspected |
|  | Diagnosis | Choice of the best imaging method: CT or MRI (including chest MRI), role of PET-CT |
|  |  | PCR for molecular diagnosis, both on BAL samples and tissues |
|  |  | New methods (sequencing, metagenomics) |
|  |  | Differentiation between Aspergillus and Mucor in the pathology laboratory. The difference between Aspergillus and Mucor hyphae. The role of dyes such as calcofluor to aid in this differentiation. |
|  | Treatment | Role of surgical treatment |
|  |  | Use of the new azoles (Isavuconazole and posaconazole) |
|  |  | Liposomal amphotericin B dosage and dose escalation |
|  |  | Adjuvant therapies, combination therapy with echinocandins |
|  | Prognosis | Mortality and clinical outcomes, according to host and clinical form |
|  |  | Importance of early diagnosis |
|  |  | Importance of surgery and control of the underlying disease |
|  | Prevention | Prevention measures for COVID-19 patients |
|  |  | Antifungal prophylaxis in high-risk patients (what drugs and which agents can it be used against) |
|  |  | Education and awareness for early recognition of associated syndromes |
| **S**  **p**  **o**  **r**  **o**  **t**  **r**  **i**  **c**  **h**  **o**  **s**  **i**  **s** | Epidemiology | Scope and epidemiological impact of S. brasiliensis: |
|  |  | The transformation of sporotrichosis into a zoonosis: feline transmission |
|  |  | Hyperendemic areas and their expansion |
|  |  | Risk factors for disseminated disease |
|  | Clinical manifestations | Different clinical forms and variation according to species |
|  |  | Disseminated forms and CNS involvement |
|  |  | Ocular and articular forms |
|  |  | Lung involvement and disease in immunosuppressed hosts |
|  | Diagnosis | Histopathology and culture (gold standard) |
|  |  | Differential diagnosis with other dermatological diseases |
|  |  | New methods (antigen/PCR) |
|  | Treatment | Itraconazole dosage and treatment time |
|  |  | Use of terbinafine as an alternative |
|  |  | Topical treatment |
|  |  | Treatment of disseminated forms |
|  | Prognosis | Recurrence and risk factors for treatment failure |
|  |  | Challenges of monitoring patients with CNS disease |
|  |  | Skin scarring / fibrosis and other sequelae |
|  | Prevention | Control and management of infected animals, especially cats. |
|  |  | Hygiene and occupational protection measures. |
|  |  | Health education for at-risk populations. |
| **C**  **r**  **y**  **p**  **t**  **o**  **c**  **o**  **c**  **c**  **o**  **s**  **i**  **s** | Epidemiology | Geographical distribution of Cryptococcus gattii |
|  |  | Ecological niches of Cryptococcus gattii and Cryptococcus neoformans |
|  |  | Cryptococcosis in hosts without apparent immunosuppression or with relative immunosuppression (age, diabetes, other comorbidities) |
|  | Clinical manifestations | Neurotropism of cryptococcosis and the need for lumbar puncture |
|  |  | Cryptococcus gattii's propensity to produce mass lesions (lung and CNS) and simulate neoplasms |
|  |  | Extra-CNS manifestations of the disease (more frequent in transplant patients). |
|  |  | Difference in the clinical presentation of cryptococcosis in AIDS versus transplant patients |
|  |  | How to recognize immune reconstitution syndrome |
|  | Diagnosis | Sensitivity and specificity of the antigen detection test |
|  |  | Advantages of CrAg compared to latex detection |
|  |  | Equivalence of CrAg and Latex antigen titers |
|  |  | Time for growth in culture |
|  |  | Mention of the use of selective culture media, such as Niger Agar |
|  |  | Use of dye in the cerebrospinal fluid to highlight the capsule |
|  |  | Specific stains in histopathology to stain the capsule |
|  |  | Influence of antifungal use on culture time and yield |
|  | Treatment | Therapy consisted of induction, consolidation and maintenance |
|  |  | Liposomal amphotericin B as the regimen of choice |
|  |  | Mention of the use of single-dose amphotericin B, as recommended in current guidelines |
|  |  | Importance of using 5-fluorocytosine in the induction regimen |
|  |  | Comment that the presence of yeasts in the blood in AIDS patients may be cryptococcosis, which does not respond to echinocandins |
|  |  | The importance of intracranial hypertension and how to manage it |
|  |  | When to start antiretrovirals in cryptococcosis |
|  |  | Management of immune reconstitution syndrome (IRS), including the use of corticoids |
|  |  | Interrupt ART when SIRS occurs? |
|  | Prognosis | Prognostic factors in cryptococcosis. These include low CSF glucose, high antigen titer, altered mental status and low CSF cellularity. |
|  | Prevention | Test and treat strategies, using antigen detection in patients with low CD4, aimed at detecting very early disease and eliminating meningitis |
|  |  | When a CrAg or LFA titer can infer disseminated disease (or CNS involvement) in preemptive therapy |
|  |  | Primary prevention with antifungals |
|  |  | How long to maintain secondary prophylaxis after treatment |
|  |  | Need for maintenance in transplant patients |
| **T**  **a**  **l**  **a**  **r**  **o**  **m**  **y**  **c**  **o**  **s**  **i**  **s** | Epidemiology | Prevalence and epidemiological association of talaromycosis with endemic areas such as Southeast Asia and southern China |
|  |  | Mention of risk factors, such as HIV/AIDS with CD4 count < 200 cells/mm³, immunosuppressive therapy, and organ transplantation |
|  |  | Discussion of the increasing identification of the disease in non-HIV and non-endemic populations due to migration and globalization |
|  |  | Presentation of updated mortality rates in treated versus untreated patients |
|  | Clinical manifestations | Accurate description of respiratory manifestations, such as fever, cough, and dyspnea, as initial symptoms |
|  |  | Mention of papular or ulcerated skin lesions as a common presentation |
|  |  | Detailed description of deep organ involvement (liver, spleen, and lymph nodes) in disseminated cases |
|  |  | Alert regarding atypical forms in immunocompetent populations |
|  | Diagnosis | The cultivation method in specific media (e.g., Sabouraud) is correctly described as the gold standard |
|  |  | Mention of the use of molecular assays, such as PCR, for rapid diagnosis |
|  |  | The book discusses the role of the galactomannan antigen as an adjunct marker in body fluids |
|  |  | Differences between talaromycosis and similar fungal diseases, such as histoplasmosis, in the differential diagnosis are addressed |
|  | Treatment | The text recommends liposomal amphotericin B as the initial therapy in severe cases, with subsequent transition to itraconazole |
|  |  | An update on the efficacy of itraconazole versus voriconazole in moderate cases is provided |
|  |  | he book discusses the role of managing underlying conditions, such as the initiation or optimization of ART in HIV patients |
|  |  | The need for prolonged maintenance therapies to prevent recurrences is mentioned |
|  | Prognosis | The impact of late diagnosis on the increase in mortality rates is adequately discussed |
|  |  | Updated data on cure rates with modern antifungal therapies are presented |
|  |  | Mention of the role of immune restoration, such as increased CD4 count, in prognosis |
|  |  | Alert regarding the risk of recurrence in patients who discontinue maintenance therapy prematurely |
|  | Prevention | he book addresses primary prevention strategies, such as fungal infection screening in immunocompromised individuals |
|  |  | Mention of the role of ART in the prevention of the disease in people living with HIV |
|  |  | Discussion of hygiene and sanitation practices in endemic regions |
|  |  | Recommendations to avoid exposure to contaminated soils or other environmental sources in endemic areas |
| **M**  **y**  **c**  **e**  **t**  **o**  **m**  **a** | Epidemiology | The book addresses the geographic distribution of mycetoma, with a focus on the mycetoma belt, particularly in Sudan and other tropical countries |
|  |  | Occupational risk factors, such as agricultural activities in endemic areas, are described |
|  |  | The text adequately differentiates eumycotic mycetoma (caused by fungi) from actinomycotic mycetoma (caused by bacteria) |
|  |  | Recent statistics on morbidity and disabilities associated with the disease are presented |
|  | Clinical manifestations | A detailed description of the insidious onset, with local swelling and formation of draining nodules, is provided |
|  |  | The book adequately addresses the drainage of grains (fungi or bacteria) as a diagnostic feature |
|  |  | Local complications, such as bone invasion and deformities, are discussed |
|  |  | Clinical differentiation between eumycotic and actinomycotic mycetoma exists |
|  | Diagnosis | The book mentions the use of direct examination and grain staining for initial identification |
|  |  | Description of imaging findings, such as ultrasound and magnetic resonance imaging, to assess bone and soft tissue involvement is provided |
|  |  | Molecular methods, such as PCR, for specific identification of causative agents are discussed |
|  |  | An adequate distinction between mycetoma and similar conditions, such as chromoblastomycosis and osteomyelitis, is made |
|  | Treatment | The text addresses the use of antifungals, such as itraconazole, for eumycotic mycetoma |
|  |  | Mention of the need for surgery in advanced cases, with removal of necrotic tissue, is made |
|  |  | The book presents evidence on the ineffectiveness of isolated antifungal agents in advanced mycetomas |
|  |  | The role of multidisciplinary management for the rehabilitation of patients with deformities is discussed |
|  | Prognosis | The text addresses the impact of early intervention in reducing permanent disabilities |
|  |  | Data on therapeutic failure in eumycotic versus actinomycotic mycetomas are presented |
|  |  | The book mentions the recurrence of mycetomas after inadequate treatment |
|  |  | Prevention strategies, such as protection in high-risk environments, are described |
|  | Prevention | Description of occupational protection strategies, such as the use of footwear and gloves, is provided |
|  |  | The text mentions educational campaigns in endemic areas to reduce skin trauma |
|  |  | Strategies for early diagnosis in vulnerable populations are discussed |
|  |  | Guidance on wound care to prevent secondary infections is provided |
| **c**  **h**  **r**  **o**  **m**  **o**  **m**  **y**  **c**  **o**  **s**  **i**  **s** | Epidemiology | Mention of the global distribution, with a focus on tropical and subtropical areas, is made |
|  |  | The book addresses risk factors, such as skin trauma in rural workers and exposure to contaminated soil |
|  |  | Recent data on the most common species, such as *Fonsecaea pedrosoi*, are presented |
|  |  | Incidence rates in vulnerable populations, such as low-income workers, are described |
|  | Clinical  manifestations | The text adequately describes verrucous, nodular, or plaque-like skin lesions |
|  |  | Mention of slow progression, with chronic local involvement and lymphedema, is made |
|  |  | The book addresses advanced cases with lymphatic or hematogenous spread |
|  |  | Description of atypical clinical forms, such as ulcerated lesions or those mimicking malignant tumors, is provided |
|  | Diagnosis | Description of the use of direct examination and cultures to identify causative organisms is provided |
|  |  | The book mentions histopathology with sclerotic bodies (“coin cells”) as a diagnostic marker |
|  |  | Modern methods, such as MALDI-TOF or genetic sequencing, for species identification are discussed |
|  |  | The text addresses differential diagnoses, such as sporotrichosis and cutaneous leishmaniasis |
|  | Treatment | The book recommends itraconazole as first-line therapy, according to guidelines |
|  |  | Mention of the role of immune restoration, such as increased CD4 count, in prognosis is made |
|  |  | The text addresses the role of combination therapy, including cryotherapy and surgical excision in localized lesions |
|  |  | Challenges in managing extensive or resistant cases are discussed |
|  | Prognosis | The impact of early versus late diagnosis on outcomes is adequately discussed |
|  |  | The text addresses the risk of complications, such as chronic lymphedema, and the importance of continuous treatment |
|  |  | Mention of treatment response rates, including refractory cases, is made |
|  |  | Strategies for prevention and reduction of associated morbidities are described |
|  | Prevention | Description of educational strategies for at-risk workers, such as hygiene practices, is provided |
|  |  | The text mentions the importance of using personal protective equipment in rural activities |
|  |  | Public health campaigns for early diagnosis and reduction of exposure are discussed |
|  |  | The book includes the importance of follow-up for treated patients to prevent recurrences |
